# Research capacity building frameworks for allied health professionals – a systematic review

**DOI:** 10.1186/s12913-018-3518-7

**Published:** 2018-09-15

**Authors:** Janine Matus, Ashlea Walker, Sharon Mickan

**Affiliations:** 1 Allied Health, Gold Coast Health, Gold Coast, Queensland Australia; 2 School of Allied Health Sciences, Griffith University, Gold Coast, Queensland Australia

**Keywords:** Research capacity building, Research culture, Research activity, Allied health, Framework

## Abstract

**Background:**

Building the capacity of allied health professionals to engage in research has been recognised as a priority due to the many benefits it brings for patients, healthcare professionals, healthcare organisations and society more broadly. There is increasing recognition of the need for a coordinated multi-strategy approach to building research capacity. The aim of this systematic review was to identify existing integrated models and frameworks which guide research capacity building for allied health professionals working in publicly funded secondary and tertiary healthcare organisations.

**Methods:**

A systematic review was undertaken searching five databases (Medline, CINAHL, Embase, AustHealth and Web of Science) using English language restrictions. Two authors independently screened and reviewed studies, extracted data and performed quality assessments using the Mixed Methods Appraisal Tool. Content and thematic analysis methods were used to code and categorise the data.

**Results:**

A total of 8492 unique records were screened by title and abstract, of which 20 were reviewed in full-text. One quantitative study and five qualitative studies were included, each of which describing a research capacity building framework. Three interconnected and interdependent themes were identified as being essential for research capacity building, including ‘supporting clinicians in research’, ‘working together’ and ‘valuing research for excellence’.

**Conclusions:**

The findings of this systematic review have been synthesised to develop a succinct and integrated framework for research capacity building which is relevant for allied health professionals working in publicly funded secondary and tertiary healthcare organisations. This framework provides further evidence to suggest that research capacity building strategies are interlinked and interdependent and should be implemented as part of an integrated ‘whole of system’ approach, with commitment and support from all levels of leadership and management. Future directions for research include using behaviour change and knowledge translation theories to guide the implementation and evaluation of this new framework.

**Trial registration:**

The protocol for this systematic review has been registered with PROSPERO. The registration number is CRD42018087476.

**Electronic supplementary material:**

The online version of this article (10.1186/s12913-018-3518-7) contains supplementary material, which is available to authorized users.

## Background

There is a burgeoning interest in strategies to enhance research capacity building for healthcare professionals. The recent Strategic Review of Health and Medical Research in Australia (2013) recommended that research should be fundamentally embedded in the health system, and that the healthcare workforce should be involved in research to drive continuous improvement [1]. Research capacity building has been defined as “a process of developing sustainable abilities and skills enabling individuals and organisations to perform high quality research” [2], or “a process of individual and institutional development which leads to higher levels of skills and greater ability to perform useful research” [3].

While there is no single agreed upon definition of “allied health” in the international literature, allied health professions are commonly grouped together by exclusion from medical and nursing/midwifery, and include but are not limited to physiotherapy, occupational therapy, speech pathology, social work, psychology, podiatry and pharmacy [4]. The benefits of allied health professionals participating in research are manifold. At a clinician level, benefits include enhanced attitudes towards research [5], an increased uptake of research evidence into practice [6, 7], and the development of critical thinking skills and a culture of evidence-based practice [8]. Clinicians who participate in research are also more likely to experience greater job satisfaction [9, 10].

At a service level, having healthcare professionals involved in research may positively influence the infrastructure and processes of client care [11]. A sound base of high quality research evidence is needed to inform the delivery of evidence-based healthcare and strategic service planning and policy making [5, 8, 10, 12, 13]. An additional benefit is being able to evaluate and demonstrate the quality and efficiency of the healthcare services being provided [6]. This is especially a priority for the allied health workforce due to the relatively low level of evidence for many allied health interventions [8, 10, 14]. Allied health professionals need to produce research evidence to demonstrate the efficiency and cost-effectiveness of their interventions and models of service delivery, or else they will increase their vulnerability to having aspects of their work delegated to traditional medical and nursing professionals, not being able to maintain current roles, diversify into new areas or expand their scope of practice [6, 8].

At a broader societal level, benefits of clinicians engaging in research include the potential of more successful translation and impact of research findings into clinical practice, thereby enhancing patient outcomes [15–17]. Indeed, having healthcare professionals involved in identifying research questions that arise from real-life problems and gaps in clinical practice and assisting with designing research methodologies may increase the likelihood that research projects will generate practical solutions which are readily translated into practice [17].

Previous research has demonstrated that allied health professionals are motivated to participate in research by intrinsic and extrinsic factors which align to these benefits. The most commonly reported motivators are to address problems in practice, build the evidence base to inform service delivery, provide the best possible care for patients and enhance their job satisfaction and career opportunities [6, 10, 18, 19].

The aim of research capacity building in a healthcare setting is to strengthen health professionals’ existing clinical expertise with complementary research skills [8]. This enables them to contribute to the production of high-quality research which advances the knowledge base of their profession, demonstrates the effectiveness of interventions, influences funding bodies, and enables evidence-based practice [8]. Building research capacity may be targeted across three different levels including foundational skills in using research (e.g. understanding how to search for, appraise and consciously apply research evidence to inform practice), participating in research (e.g. assisting with participant recruitment and data collection) and leading research (e.g. developing research protocols and applying for funding).

Allied health professionals have been reported to have a high level of interest in undertaking research [20–22]. However, despite their interest and the recognised benefits, allied health research engagement remains limited due to a number of challenges and barriers including a lack of time and funding, other work roles taking priority, a lack of research skills and a lack of support from managers and colleagues [10, 19]. As building allied health research capacity has been recognised as a priority [10], a range of different research capacity building approaches have been recommended and implemented across publicly funded healthcare organisations in Australia [9, 23] and internationally [8, 24].

Most of the extant literature describes single-strategy research capacity building initiatives, interventions or programs. Some of these strategies have been focussed at the level of individuals and teams, such as identifying those clinicians who express motivation and intention to do research and those who are seeking a challenge, improved job satisfaction or increased professional development opportunities [10, 19, 22] and providing these clinicians with protected time, education and training, resources and mentoring from more experienced researchers [10, 18, 22, 25–27]. For example, a research internship model for podiatrists resulted in increased research output, as measured by the number of abstracts, publications and further research training [28].

Dedicated research leadership/facilitator or conjoint positions have been found to be associated with increased organisation and team domain scores on the Research Capacity and Culture tool, as well as increased research skills and outputs [7, 29, 30]. Similarly, academic-practice partnerships have been reported as an important strategy for increasing research capacity, engagement and output [10, 27, 31, 32]. For example, a large proportion of research outputs by clinical staff within one large publicly funded health service were the result of work led by, or in collaboration with, academic partners [27].

Strategies which have been implemented at the level of the organisation include embedding research activities in strategic plans, visions, missions and values, developing targets or key performance indicators (KPIs) for research [19] and role descriptions to attract research interested and active applicants [10]. Organisation level strategies also include incorporating research into clinical roles, increasing funding for appropriate backfill of clinical positions, supporting staff with joint clinical and academic appointments [6] and creating opportunities to engage in research through secondment [6, 8, 12, 27]. It has been suggested that organisations may benefit from strategically prioritising funding for those projects which have the greatest potential to directly impact on patient care [8].

Some authors have recognised that a single strategy approach is not sufficient, but that a “whole of organisation approach” or “whole of system approach” is required for building research capacity and culture in allied health [10, 12, 33–35]. A recent rapid review of allied health research frameworks has recommended multiple strategies across individual, organisational and policy levels to embed a culture of allied health research into healthcare services [36]. Authors have suggested that strategies are interlinked and interdependent, such that strategies implemented at one level can have an impact on other levels. Therefore, the use of coordinated and integrated multi-level strategies at individual, team, organisational and system levels has been recommended [18, 25, 33, 37]. However, there currently is no single framework, model or set of recommendations to guide research capacity building approaches for allied health professionals in publicly funded secondary or tertiary healthcare settings.

The aim of this systematic review was to identify, appraise and synthesise existing models and frameworks which describe integrated and practical approaches to research capacity building for allied health professionals in publicly funded secondary or tertiary healthcare organisations. This review intended to search for both models and frameworks, the most common methods of conceptualising combinations of strategies. A model usually describes and guides the process of implementing an intervention, including a temporal sequence of steps, stages or phases of the process. In contrast, a framework usually identifies the hypothesised factors which may influence an outcome without describing the process for achieving this outcome. A framework may also provide a structure for planning and evaluating interventions. Neither models or frameworks necessarily address the causal mechanisms of change [38]. The protocol for this systematic review has been registered with PROSPERO. The registration number is CRD42018087476.

## Methods

### Search methods

In collaboration with authors AW and JM, a senior librarian developed a detailed search strategy in the following five electronic databases: Medline (Ovid), Embase (Elsevier), CINAHL (Ebsco), AustHealth (Informit) and Web of Science (Clarivate Analytics). Terms and synonyms relating to research capacity building, allied health, hospital and healthcare service/organisation, model and framework were used. Database searches were conducted on the 19th and 27th June 2017. An example of the search strategy used in Medline is found in Additional file 1. The search terms were adapted as required to search the other four databases. Reference lists of included articles were additionally reviewed. Where full-text articles were not available, or clarification was required, one of the authors (JM) contacted the study authors to request the relevant information.

### Study inclusion and exclusion criteria

The eligibility criteria for this study are described in Table 1 below. As the purpose of this systematic review was to address an identified need for evidence-informed allied health research capacity building approaches in a publicly funded secondary and tertiary healthcare organisation, the inclusion and exclusion criteria have been tightly scoped to reflect this. Only studies published in the English language and between January 2005 and June 2017 were included. These decisions were made in the interest of resourcing feasibility.

**Table 1:** Inclusion and exclusion criteria

	Inclusion criteria	Exclusion criteria
1. Consists of a suite of research capacity building approaches.	Yes	No. Single strategies and interventions were excluded.
2. Specifically targets one or more allied health professions’ capacity to perform research.	Yes	No. Approaches which target only nursing or medical professionals were excluded. Approaches which only target health professionals’ capacity to use research were excluded.
3. Includes a rigorous peer-reviewed evaluation component.	Yes	No. Theoretical, expert opinion and conference papers and grey literature were excluded.
4. Developed for a publicly funded secondary or tertiary healthcare setting, including hospital, outpatient and/or community-based services.	Yes	No. Approaches developed exclusively for primary and private healthcare settings were excluded.

### Study selection

Search results and additional references were collated into a reference database (Endnote) and any duplicates deleted. All titles and abstracts were independently screened by two authors to identify studies that potentially met the eligibility criteria. Full text copies of these articles were retrieved and independently assessed for eligibility by two authors. Disagreements were resolved by discussion and consensus agreement, and if required, input from a third author.

### Data extraction and quality assessment

Data were independently extracted and analysed by two authors, using a data extraction form developed to include information pertaining to study location, participant demographics, purpose, definition of research capacity building, methodology and study design. Disagreements were resolved through discussion and consensus agreement.

The extent to which each study is likely to be influenced by bias was independently evaluated by two authors using the Mixed Methods Appraisal Tool (MMAT). This tool was designed to concomitantly appraise the methodological quality of studies with diverse designs including qualitative, quantitative and mixed methods research [39]. Two consistent screening criteria are complemented by four methodological criteria for each study design.

## Results

**Fig. 1:**
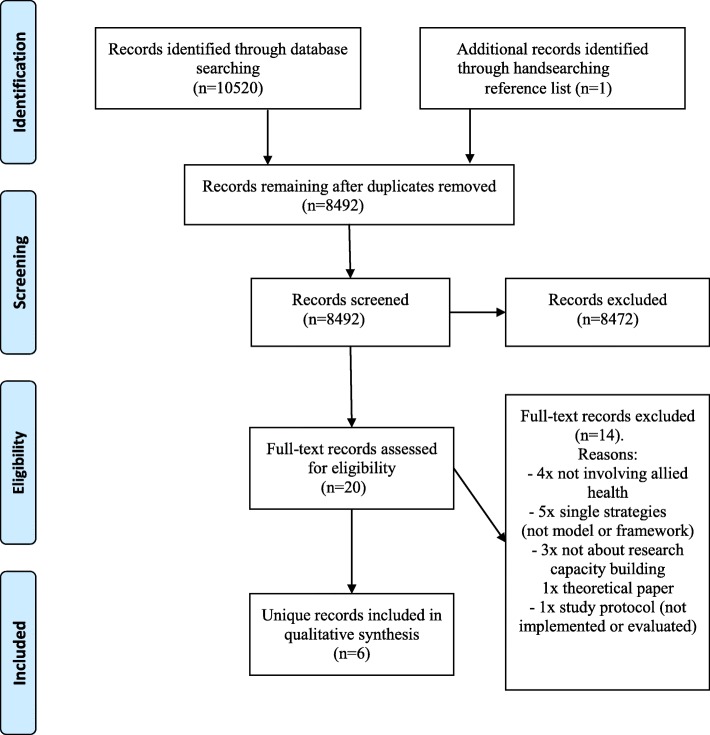
Flow diagram of process to identify eligible studies

A total of 8492 unique records were assessed for eligibility by screening titles and abstracts. Of these, 20 were reviewed in full-text and six were included in the review [9, 29, 33, 37, 40, 41]. Figure 1 illustrates the number of studies which were screened based on title/abstract and full-text, with reasons for exclusion documented.

A total of one quantitative and five qualitative studies were included. Studies originated in Australia (*n* = 4) and the UK (*n* = 2). All studies defined research capacity as the ability to engage in, perform or carry out quality research. All six studies met the definition of framework rather than model. The studies varied in terms of the composition of their frameworks and in the way that these had been developed, implemented and evaluated. Each framework describes number of research capacity building approaches. Refer to Table 2 for a description of the included studies.

**Table 2:** Description of included studies

Study	Population	Purpose and intervention	Study design	Data collection and analysis	Research capacity building approaches described in each framework	Structural levels	Assumptions	Research capacity building definition
Bamberg, Perlesz, McKenzie & Read, 2010 [40]	Australia: Large not-for-profit community healthcare organisation	Build and enhance the capacity of a community health service to conduct their own research and evaluation	Qualitative (cooperative inquiry action research design)	Semi-structured interviews, meeting observations, reflective journals Documented changes in: - staff requests for assistance - confidence and knowledge - policy and procedures Thematic analysis	1. Dissatisfaction with the status quo 2. Knowledge and skills 3. Available resources 4. Available time 5. Rewards or incentives 6. Participation is expected/encouraged 7. Commitment by those involved 8. Leadership	None specified	Ely’s ‘8 conditions for implementing innovation’ model support the successful implementation of a framework to produce change	None reported
Cooke, Nancarrow, Dyas & Williams, 2008 [29]	UK: ‘Designated research teams’ from primary and community care, funded by a large Research Development Unit, including social workers, pharmacists and podiatrists	Implement and evaluate the ‘designated team approach’ for 6 clinical teams using funding, mentorship and expert support	Qualitative Evaluated against Cooke (2005)’s framework of 6 principles of research capacity building	Process and outcome measures collected on context, activities, experiences, outputs and impacts of interventions Narrative summary of results	1. Development of appropriate research skills to apply in practice 2. Development of linkages, partnerships and collaborations 3. Support of research that is ‘close to practice’ 4. Appropriate dissemination 5. Appropriate infrastructure 6. Elements of continuity and sustainability	Individual Team Organisation Supra-organisational	Research capacity building initiatives may occur within, and across, structural levels	‘A process of individual and institutional development which leads to higher levels of skills and greater ability to perform useful research’ (Trostle, 1992, p.1321) [3]
Golenko, Pager & Holden, 2012 [33]	Australia: Large public healthcare organisation Nine AH senior managers of five hospital and community teams	Develop a thematic model to promote and enhance allied health research capacity at an organisational level	Qualitative	Semi-structured in-depth interviews with senior AH managers. Conceptual and relational analysis using NVivo	1. Whole of organisational approach and support from senior managers 2. Structures, processes and systems 3. Partnerships and collaborations 4. Dedicated research centres, units and positions	Individual Team Organisation External environment	The organisation is the critical link in creating synergy across the 4 levels of research capacity building	‘A process of individual and institutional development which leads to higher levels of skills and greater ability to perform useful research’ (Trostle, 1992, p.1321) [3]
Holden, Pager, Golenko, Ware & Weare, 2012 [37]	Australia: Large public healthcare organisation AHPs working in a mixture of hospital and community settings	Evaluate the impact of a multi-strategy team based research capacity building intervention	Quantitative Non-randomised matched-pairs trial	Research Capacity and Culture Tool (RCC) Linear regression analysis using a random effects mixed model	1. Tailored research skills training programs 2. Ongoing mentoring 3. Writing bursaries 4. Financial support 5. Research fellowships 6. Infrastructure support	Individual Team Organisation		Research capacity building develops individuals and institutions to higher levels of skill and ability to conduct quality research
Hulcombe, Sturgess, Souvlis & Fitzgerald, 2014 [9]	Australia: Public hospital and health services Allied health, oral health and scientist practitioners	Develop an organisational research capacity building framework	Descriptive qualitative Evaluation strategy based on program logic methodology	Literature review, stakeholder consultation Changes in no. of: - new researchers - research proposals - applications for grant funding - peer reviewed publications Qualitative analysis	1. Leadership and governance 2. Supporting researchers 3. Translation of evidence into practice			‘A funded, dynamic intervention… to augment ability to carry out research or achieve objectives in the field of research over the long-term, with aspects of social change as an ultimate outcome’ (Condell & Begley, 2007, p.273) [47]
Whitworth, Haining & Stringer, 2012 [41]	UK: Speech and language therapists (SLT) and academics in healthcare and higher education	Develop a model for building research capacity	Historical descriptive with qualitative evaluation	Literature review Evaluation of - funded research activity - skill development - change in research culture, increase in research confidence Qualitative analysis	1. Whole system approach 2. Accommodating diversity 3. Reducing barriers 4. Enabling collaboration 5. Providing feedback and mentoring 6. Facilitating networking	Research conscious Research participative Research active	The need to embed, influence and contribute to research is a common driver for all health and social care professional groups	Increased capability to engage in research is fundamental to translation of research into practice and to support excellence in healthcare research

### Risk of bias within studies

All studies had a clear research question or objective and collected relevant data to address it. Studies varied in their methodology and in how comprehensively this was reported. Based on their MMAT scores, all six studies were judged to be of appropriate and comparable quality to be included in a narrative synthesis. Refer to Table 3 below for a descriptive summary of the methodological quality and risk of bias of each study using the MMAT criteria.

**Table 3:** Quality assessment of studies

	Screening (yes/no/unclear)		Methodological quality criteria - Quantitative non-randomised (yes/no/unclear)			
Study	Clear research question	Relevant data	3.1 Selection bias minimised	3.2 Appropriate measurements	3.3 Comparable participants	3.4 Outcome data for 80% or above
Holden et al., 2012 [37]	Yes	Yes	Yes	Yes	Yes	Unclear
	Screening (yes/no/unclear)		Methodological quality criteria - Qualitative (yes/no/unclear)			
Study	Clear research question	Relevant data	1.1 Relevant sources of data	1.2 Relevant data analysis	1.3 Consideration of context	1.4 Consideration of researchers’ influence
Bamberg et al., 2010 [40]	Yes	Yes	Yes	Yes	Yes	Unclear
Cooke et al., 2008 [29]	Yes	Yes	Yes	Yes	Yes	Yes
Golenko et al., 2012 [33]	Yes	Yes	Yes	Yes	Yes	Yes
Hulcombe et al., 2014 [9]	Yes	Yes	Yes	Unclear	Yes	Unclear
Whitworth et al., 2012 [41]	Yes	Yes	Yes	Unclear	Yes	Unclear

### Data analysis

Qualitative analysis was used to synthesise findings. Initial steps of the qualitative analysis involved an attempt to directly compare the overarching research capacity building approaches described in each framework. The total number of approaches was 33, ranging from three to eight per framework. Please refer to Table 2 for details of these approaches. However due to differences in terminology and classification, it was not possible to compare these approaches directly. Due to variations in their purpose, content and theoretical design, no single framework was able to explain all of the approaches included in the others.

Instead, a content analysis method [42] was used to code and categorise the individual components of each approach (total number = 162), which were defined for the purpose of this review as the discrete strategies and conditions within each approach that were found to be conducive to research engagement and capacity building. These coded components were then grouped according to their frequency and emerging patterns of similarity and consistency in their content, both within and across the frameworks.

Next, an inductive thematic analysis was undertaken following the phases described by Braun & Clarke [43]. Phases included searching for underlying patterns of meaning among the coded components and groups of components, generating preliminary themes, reviewing the themes, and naming the themes [43]. This process was recursive and made use of thematic mind maps to explore relationships between the codes and themes. Each preliminary theme was reviewed to ensure that its included codes formed a coherent pattern. Some themes were consolidated while others were subdivided or reworked to ensure both internal homogeneity and external heterogeneity.

Ultimately, three interconnected and interdependent themes were identified as being essential for building research capacity. These are ‘supporting clinicians in research’, ‘working together’ and ‘valuing research for excellence’. These themes are supported by 17 subthemes. Two authors contributed independently to the analysis and met regularly to challenge each other’s assumptions and cross-check the validity of the preliminary and final themes to help maintain trustworthiness, credibility and accountability of the findings [44]. All authors agreed on the final themes. Please refer to Table 4 for an overview of the final themes and subthemes and to Additional file 2 for a detailed list of all coded and categorised components which are presented as lists of strategies linked to each subtheme.

**Table 4:** Overview of themes and subthemes

Supporting clinicians in research	Working together	Valuing research for excellence
- Education and training - Opportunities to get involved - Research friendly workplace - Mentoring/coaching - Access to resources - Protected time and funding - Reward and recognition - Support to undertake post-graduate study including HDR - Skill mix of teams	- Collaborations and partnerships with other teams, services and organisations - Shared purpose and drivers - Coordinated approach including team-based research projects - Shared expertise	- Visible support for research - Research as core business - Prioritisation of research that is ‘close to’/relevant to practice - Integration of local research findings back into practice

### Theme 1: supporting clinicians in research

Research capacity is built by supporting allied health professionals to develop research knowledge, skills and confidence. A range of strategies were documented in the literature and have been summarised into the following sub-themes:

- relevant education and training for undertaking aspects of the research process such as writing grant and ethics applications;
- opportunities to learn and apply skills in practice including assisting with collecting data for research projects, identifying research questions, leading small research projects and participating in journal clubs;
- a research friendly workplace which accommodates and values individual clinicians’ research interests, motivations, abilities, time commitments and career paths;
- mentoring and coaching from more experienced researchers;
- access to resources including library, software, desk and computer use;
- protected time and funding including support to apply for external research funding;
- a system of reward and recognition through the provision of greater career opportunities, research career pathways and financial incentives;
- support to undertake formal post-graduate study including higher degrees by research (HDR);
- mix of clinicians with different levels of research skills within each team.

### Theme 2: working together

Research capacity building is supported and enhanced when allied health professionals work with others in order to exchange ideas, knowledge, skills and resources and build a ‘critical mass’ of research-active staff. This may be achieved by developing:

- strategic collaborations, partnerships, linkages and networks within and between teams, services and organisations including universities and industry;
- shared purpose / drivers for research;
- coordinated and team-based projects;
- opportunities to share research expertise with others in the team and wider networks.

### Theme 3: valuing research for excellence

To build research capacity in a healthcare setting, allied health professionals need to feel that their engagement in research is valued as contributing to excellent service delivery. This may be fostered by:

- demonstrating visible support of and endorsement of research at the management level, including developing structured processes and systems for research and restructuring clinical roles to include some time for research;
- prioritising research as part of a health service’s core business by including research in the service’s vision, mission, strategic plans, key performance indicators and role descriptions;
- prioritising research projects which are close to / relevant to practice and in line with strategic priorities,
- reporting, disseminating and applying locally developed research findings to inform practice.

## Discussion

The findings of this systematic review have been synthesised to develop a succinct and integrated framework for research capacity building which is relevant for allied health professionals working in publicly funded secondary and tertiary healthcare organisations. Three themes (‘supporting clinicians in research’, ‘working together’ and ‘valuing research for excellence) and 17 subthemes have been identified. Each subtheme is linked to a number of strategies which may be implemented at individual, team, organisational and policy levels as part of the ‘whole of system’ approach which has been recommended in the literature [12, 33, 36, 45]. Although attempts were made to categorise strategies according to these structural levels, it was subsequently recognised that many strategies are applicable at more than one level. For example, for research to be considered part of core business, it needs to be valued by individual clinicians and by all levels of management across teams and the organisation and recognised within policy. This new framework consolidates many single-strategy research capacity building initiatives, interventions or programmes described in the literature, and provides further evidence to suggest that they are interlinked and interdependent and therefore benefit from being delivered in an integrated way to ensure maximum impact.

Although this review searched for both models and frameworks, only frameworks were found. It seems that frameworks are inherently better suited to guide research capacity building, because they do not include a clear linear process for how research capacity building interventions should be implemented. A number of factors appear to influence the outcomes of research capacity, culture and engagement and are useful for guiding the design and evaluation of interventions. However, the way in which interventions are implemented is highly dependent on context, such as the specific strengths, weaknesses, interests, needs and priorities of each individual, team and organisation.

A fundamental concept which was identified across all three themes is the importance of commitment and multi-faceted support from all levels of leadership and management. A research culture has been described as “an environment within an organisation that enables and supports research to generate new knowledge and opportunities to translate evidence into practice” [18] and has been reported to be essential for building research capacity [19, 33]. Previous studies have found that senior management and leadership support for research appears to have a significant impact on an organisation’s research culture [7, 20, 35, 36, 46] and individual health professionals’ engagement in research [29, 31]. The findings of this review further emphasise that in order to build and sustain research engagement, leaders and managers should recognise the benefits of having research-active practitioners in the workforce and consider research to be part of their core business alongside clinical practice [8, 19, 27]. Another implication is the importance of investing in collaborations with internal and external partners, mentors and colleagues who can support clinicians to undertake research within their existing roles, which is consistent with previous recommendations in the literature [22, 25, 32].

### Limitations

As the purpose of this systematic review was to inform a broader research capacity building project being conducted in a large publicly funded secondary and tertiary healthcare organisation, a decision was made to tightly scope the search strategy and eligibility criteria to maximise relevance to our context. A limitation of this decision is that the results may not be transferable to other contexts.

Overall, there is a paucity of published evidence-informed research capacity building models and frameworks which are suitable for allied health. Moreover, the extant literature about research capacity building is poorly indexed using variable search terms. For example, different terms and definitions are used to describe models and frameworks. As a result, it was challenging to construct a search strategy which captured all relevant articles. There is a need for a better taxonomy of terms relating to research capacity building to assist with indexing, searching and identifying relevant articles.

Another limitation was that the term ‘primary care’ is inconsistently used in the literature. Although this term usually refers to settings where clinicians work independently and have first contact with clients, through hand searching of the literature, we have found three articles which use the term ‘primary care’ but refer to a population which meets this study’s criteria of secondary care. Therefore, it is possible that other studies have been missed because they were not captured by the search strategy.

## Conclusions

This systematic review developed a succinct and integrated framework for allied health research capacity building. This framework may be used to inform and guide the design and evaluation of research capacity building strategies targeting individuals, teams, organisations and systems. This framework provides structure in terms of specific strategies which can be monitored using process and outcome measures to determine short- and long-term impacts. Future directions for research include using behaviour change and knowledge translation theories to guide the implementation and evaluation of this framework. Another opportunity is to evaluate the transferability of this framework to other healthcare professions and settings.

## Additional files

Additional file 1: Search strategy. (DOC 25 kb)

Additional file 2: List of coded components mapped against themes and subthemes. (DOCX 34 kb)

## Abbreviations

MMAT: Mixed Methods Appraisal Tool
