# Supplementary material for: Research capacity building frameworks for allied health professionals – a systematic review
Source: BMC Health Serv Res. 2018 Sep 15;18:716. doi: 10.1186/s12913-018-3518-7 (PMC6139135; doi:10.1186/s12913-018-3518-7)
Supplement: Supplementary file 1 — Search strategy. (DOC 25 kb) [file 12913_2018_3518_MOESM1_ESM.doc]

Additional File 1: Example of search strategy for Medline (Ovid)

1 allied health occupations/ or audiology/ or occupational therapy/ or physical therapy specialty/ or speech-language pathology/ or technology, radiologic/ or exp dentistry/ or dietetics/ or optometry/ or podiatry/ (423914)

2 allied health personnel/ or audiologists/ or exp dental staff/ or exp dentists/ or nutritionists/ or occupational therapists/ or optometrists/ or dental staff, hospital/ or pharmacists/ or physical therapists/ (46113)

3 exp Social Work/ (16970)

4 Social Workers/ (185)

5 speech therapy/ (5896)

6 Music Therapy/ (2972)

7 exp Dental Auxiliaries/ (12842)

8 Orthoptics/ (1827)

9 (allied health or physiotherap* or occupational therap* or social work* or (speech adj3 (therap* or patholog*)) or dietitian* or dietician* or pharmacist* or exercise physiologist* or podiatrist* or sonographer* or medical radiation scientist* or music therap* or oral hygienist* or dental hygienist* or dentist* or optometrist* or prosthetist* or orthoptist*).tw. (158103)

10 exp Health Personnel/ (451665)

11 (health* adj2 (professional* or personnel or practitioner* or workforce or worker*)).tw. (128391)

12 Health Services/ (23678)

13 health service*.tw. (91972)

14 or/1-13 (1139241)

15 Capacity Building/ (1584)

16 (capacit* adj2 (build* or develop*)).tw. (8093)

17 (structur* adj2 initiat*).tw. (951)

18 Program Development/ (26973)

19 Program Evaluation/ (56514)

20 Financing, Organized/ (6468)

21 or/15-20 (90270)

22 Research Personnel/ (13961)

23 exp Research/ (556007)

24 Research Support as Topic/ (22177)

25 Health Services Research/ (35374)

26 research.tw. (1155654)

27 or/22-26 (1552578)

28 21 and 27 (27580)

29 Translational Medical Research/ (8126)

30 ((knowledge or research) adj2 translation*).tw. (10785)

31 (research* adj3 (engag* or enhanc* or uptak* or capacit* or capabil* or participat* or outcome* or cultur* or activ* or strateg*)).tw. (51818)

32 (research* adj3 (collaborat* or partner* or individual* or position* or leader* or team* or organi?ation* or supra-organi?ation* or network*)).tw. (36832)

33 or/28-32 (122072)

34 framework*.tw. (203856)

35 models, theoretical/ or models, organizational/ (152985)

36 (model or models).tw. (2204600)

37 or/34-36 (2417149)

38 14 and 33 and 37 (4638)

39 limit 38 to (english language and humans and yr="2005 -Current") (3017)

40 remove duplicates from 39 (2821)
