# Supplementary material for: Research capacity building frameworks for allied health professionals – a systematic review
Source: BMC Health Serv Res. 2018 Sep 15;18:716. doi: 10.1186/s12913-018-3518-7 (PMC6139135; doi:10.1186/s12913-018-3518-7)
Supplement: Supplementary file 2 — List of coded components mapped against themes and subthemes. (DOCX 34 kb) [file 12913_2018_3518_MOESM2_ESM.docx]

Additional file 2: List of coded components mapped against themes and subthemes

| **Supporting clinicians in research** |
| --- |
| Education and training   - Build awareness of the benefits of engaging in research activities [40]. - Undertake regular research training needs assessments [29]. - Provide a research skills training program [40]. - Provide flexible learning packages and training schemes [29]. - Provide training which is inclusive and appropriate to the needs of different professionals, shaped around their skills, backgrounds and needs [29]. - Provide training using a range of methodologies and examples to support appropriate learning [29]. - Coordinate access to training, and funds for training (use of training funds) [29]. - Provide access to research skills training [33]. - Provide opportunities for staff to develop research skills, e.g. through scholarships and workforce development programs [33]. - Provide tailored research skills training programs [37]. - Develop and advocate for (state-wide) programs that initiate and support health practitioner research and skills development [9]. - Accommodate the different research needs of individuals through targeted but highly inclusive events that focus on developing research skills [41]. - Draw on existing networks and higher education opportunities for skill development [41]. - Develop an interactive website to inform, educate and disseminate information about research [41].   Opportunities to get involved   - Encourage as many staff members as practical to participate in building research capacity within the organisation [40]. - Encourage and provide opportunities for all staff to get involved in doing research [29]. - Encourage individuals to be involved in generating research questions and contribute to developing research agendas/priorities [29]. - Create opportunities for continued application of research skills in practice, to sustain skills [29]. - Enable opportunities to extend skills and experience (concept of a “research escalator”) [29]. - Provide access to opportunities to apply research skills [33]. - All staff are expected to have an awareness of research in the workplace and the skills to seek, critique and use evidence as part of their daily practice. Depending on the stage of their interests, abilities, motivation, time commitments and service needs, staff members are also encouraged to be involved in identifying research questions based on practice issues, helping with participant recruitment or data collection. A few staff members may lead research projects [41]. - Support staff at all levels (from new grads to senior managers) to turn ideas into research projects [41]. - Supporting individuals with one-on-one meetings with academics to draw out research questions from practice and link practitioners directly into opportunities with students [41]. - Promote journal club as being not only as a forum for reading and discussing new research findings, but also as a tool to support research projects through critically appraising relevant literature [41]. - Strategically feed final year students’ research projects into service priorities and larger research projects (helps the emergence of clearer research questions for clinicians who are in the early stages of forming their ideas) [41]. - Optimise flow of information about upcoming conferences and fellowships. Often managers are more aware of research information than practitioners [29].   Research friendly workplace   - Consult staff members regarding that they think is necessary to build research capacity [40]. - Promote the everyday application of critical thinking skills, as these skills are foundational to doing research [29]. - Give research motivated clinicians the opportunity to enhance their research knowledge and expertise [29]. - Offer and support secondment opportunities to build research skills [29]. - Accommodate and value the diversity of individuals’ research needs, interests, motivations, abilities, time commitments and career paths [41]. - Support joint academic-practice appointments [41].   Mentoring/coaching   - Research skill development can be supported through mentorship and supervision [29]. - Engage with mentoring/support/supervision opportunities [29]. - Support research mentoring/supervision/coaching of research projects [29]. - Continue to engage with supervision/mentoring relationships over time [29]. - Develop structures/processes for research mentoring (e.g. mentoring agreements) [29]. - Match novice researchers with more experienced researchers. The more experienced researchers can assist less experienced researchers with writing ethics, governance and competitive grant applications [29]. - Seek out academic support for research projects [29]. - Dedicated research leadership/facilitator or conjoint positions [29]. - Establish dedicated research positions / research fellows / development officers [29]. - Ongoing mentoring or coaching for staff while they conduct their research projects. Coaching allows for learning through practical experience to promote self-efficacy to conduct projects [40]. - Mentoring in individual or group format [40]. - Support from a Research and Evaluation to discuss and seek advice regarding the design, analysis and reporting of research projects [40]. - Mentors, role models and research champions [33]. - Research mentoring to increase individual research knowledge and skills [41]. - Mentors can help to draw out research questions from practice, provide support and detailed feedback through each step of the process, to ensure a well-designed and executed research project [41]. - Provide ongoing research mentoring, e.g. to assist with writing grant applications [37]. - Create research officer positions to attract experienced researchers to mentor and drive research [33]. - Develop dedicated research centres, units and positions with associated allocated funding [33]. - Research facilitator, e.g. to liaise with Ethics Committees and navigate different funding streams [41].   Access to resources   - Provide access to infrastructure and resources such as libraries and computer software [33]. - Access to infrastructure e.g. research software, desk and computer use [37]. - Develop a site on the organisation’s intranet that is solely devoted to research and evaluation, to distribute necessary resources (simple and easily accessible), e.g.  - access to research software. - information regarding how to design, collect and analyse research and evaluation data (e.g. questionnaire templates). - how to write reports and articles for publication. - access to electronic databases. - other links to useful internet sites concerned with research. Resources are monitored and get updated according to emerging requirements [40]. - Infrastructure to help direct staff to research support and resources [29]. - Interactive website to inform, share, educate and disseminate information [41].   Protected time and funding   - Backfill individual staff members’ clinical work and/or additional funding [40]. - Pool funds to employ a research assistant who can assist staff members to conduct research projects [40]. - Provide funding for research [29]. - Provide funding for research training [29]. - Increase availability and use of training funds [29]. - Protect research time and backfill arrangements as well as funding to support this [29]. - Responsive access to, and use of, local funding [29]. - Optimise flow of information about upcoming funding [29]. - Provide access to funding [33]. - Develop systems that allow clinicians to take time off line to do research [33]. - Provide quarantined time for research (e.g. one day per week for one person from each team) [37]. - Provide some financial supports when grant applications are unsuccessful [37]. - Provide access to financial and human resources. - Increase access to funding for research e.g. supporting individuals and teams by providing small amounts of (in-kind) funding to backfill clinical time. (Initial small grant can get projects started, e.g. small scale /pilot projects, reviewing the literature, preparing larger competitive grant applications - and allow staff to build sufficient knowledge/skills/confidence [41]. - Strategic use of supernumery resources (e.g. student therapists) either to assist with doing research (as in honours students) or supporting backfill of clinical caseloads [41].   Reward and recognition   - Identify and maximise intrinsic rewards e.g. personal satisfaction in succeeding or doing something different from daily routine and practice [40]. - Provide extrinsic rewards e.g. financial, greater recognition, more resources and greater professional opportunities [40]. - Recognition and reward of research skills and achievements [29]. - Organise local team or organisation-wide conferences for staff to present papers on their current research and evaluation initiatives to other staff (to provide acknowledgement for efforts) [40]. - Encourage and support staff to attend external conferences (extrinsic reward through the recognition of their research project results) [40]. - Encourage research skill development in context of career development [29]. - Establish and support access to research career pathways in organisations [29]. - Recognition for research as a career path [33]. - Research career path framework [33]. - Promote research career opportunities [33]. - Promote recognition for research [33]. - Develop research career pathways [33]. - Provide incentives for doing research, e.g. it being beneficial for your career path [33]. - Establish and promote health practitioner research career pathways.   Support to undertake post-graduate study including higher degrees by research   - Support staff members to complete postgraduate study / RHD to further develop research skills [41]. - Increase incentives to acquire research qualifications [33].   Skill mix of teams   - Consider research skill mix across teams [29]. - Utilise existing research capacity which has already been built, e.g. by engaging those staff members who have previously gained research skills to help more novice researchers [29]. - Find an appropriate place to position the person with expertise within the team/organisation [29]. |

| **Working together** |
| --- |
| Collaborations and partnerships with other teams, services and organisations   - Linkages, partnerships and collaborations enhance and are integral to research capacity building, as they are the mechanisms by which research skills and practice knowledge is developed, exchanged and enhanced (i.e. from generating research ideas to carrying out the entire research process) [29]. - Work inter-professionally [29]. - Develop networks with other teams, including inter-professional research networks [29]. - Work with other organisations [29]. - Make links with universities [29]. - Establish conjoint appointments between health service and universities [29]. - Work with funding bodies [29]. - Building trust between different groups and individuals can enhance the information and knowledge that is exchanged [29]. - Linkages that can enhance research capacity building can exist between: - universities and practitioners. - novice and experienced researchers. - different professional groups. - different health care provider sectors - service users, practitioners and researchers. - researchers and policy makers. - different countries  - health and industries [29]. - Focus on building and maintaining collaborations over time [29]. - Partnerships with other services [40]. - Form partnerships to increase collaboration and sharing of resources and knowledge [33]. - Build internal links and collaborations between community and hospital settings and the different professions using a multidisciplinary approach [33]. - Develop a coordinated and multi-disciplinary approach to attain a critical mass of research-active staff and to enhance learning and development [33]. - Develop appropriate links with external partners [33]. - Build strong external partnerships with other organisations (especially universities), which can assist with providing access to experienced researchers, research skills training and opportunities to apply research skills, access to infrastructure and resources such as libraries and computer software and access to funding [33]. - Establish partnerships through:  - co-funded research projects and   - collaborative research positions [33]. - Partnership approach is an important element to support research capacity building and enhancement of a research culture [9]. - Develop partnerships (e.g. between allied health professions, between facilities, across public health services and between public health services and universities) [9]. - Build and maintain strategic partnerships and collaborations [9]. - Create multidisciplinary opportunities [9]. - Intra- and inter-disciplinary collaborations [41]. - Practice-academic partnerships which enable the reciprocal exchange of ideas, knowledge and skills between practitioners, academics and students [41]. - Share and link practice-driven research ideas with the perspectives and skill base of academic partners [41]. - Work together to turn practice-driven research ideas into research projects which are aligned to service needs, have testable hypotheses and appropriate designs/methodologies [41]. - Multi-centre projects [41]. - Collaborations with industry and the private sector [41]. - Collaboration with national research initiatives [41]. - Use dedicated Research Facilitator positions to support the collaboration between clinicians and academics, e.g. through running joint events and preparing joint research applications [41]. - Develop partnerships through shared research events where practitioners can discuss ideas with academics [41].   Shared purpose and drivers   - Coordinate research programs/agendas between health organisations and universities [29]. - Identify and understand the range of strategic drivers for research operating at individual and organisational levels. This is essential for determining priorities, ensuring motivation, setting up mechanisms for working together, accessing funding and implementing outcomes [41]. - Develop a shared vision and common values underpinning collaborations/ partnerships, based on mutual trust, inclusivity, transparency and respect [41]. - Get equal commitment from all partners – to ensure reciprocal exchange of knowledge and skills [41]. - Share ownership of research ideas with partners [41]. - Commit time to the early stages of the process of developing collaborative projects [41]. - Identify proposed outcomes and impact of collaborative projects early on, e.g. impact on practice and patient outcomes [41]. - Specify measurable outcomes and impacts for practice at the outset, and link these to the strategic aims of the partner organisation/s. Making strategic links will enable greater progress towards achieving the goals of the integrated collaboration [41]. - Coordinate of research programs with other organisations incl. universities [41]. - Link up partners who are geographically close and have common local drivers [41].   Coordinated approach including team-based research projects   - Develop a coordinated approach to research rather than individual research activities occurring in isolation [33]. - Support research which requires a coordinated approach [33]. - A team-based approach can achieve an appropriate mix of skills and enhance knowledge sharing [33]. - Individuals are more likely to engage in research if they are part of a team that is undertaking research or has a strong culture of research, especially if they are led by a supportive manager [29]. - Conduct a small research project as part of a team, using protected time, skills training and team mentoring [37]. - Research practice networks.   Shared expertise   - Share knowledge within teams, partnerships, collaborations and networks – e.g. sharing expertise within the context of a project, to build skills of novice researchers [29]. - Share new skills with others [29]. - Match novice researchers with more experienced researchers [29]. - Staff members to share their research interests and findings with others, causing enthusiasm to “snowball” to other staff members [40]. |

| **Valuing research for excellence** |
| --- |
| Visible support for research   - Management commitment and endorsement of research [40]. - Visible/tangible support for research (not solely verbal) – e.g.  - inclusion of research and in staff position descriptions. - inclusion of research in the orientation of new staff members. - inclusion of research in program planning. - employment of a part-time research assistant [40]. - Support from senior managers appears to have a significant impact on individual health professionals’ skills and confidence to engage in research [29]. - Visible support for research (e.g. funding for clinical backfill, designated research positions, protected research time), research structures and processes [29]. - Team leaders/managers are involved in research (e.g. directly leading or facilitating) [29]. - Support from team leaders and managers is demonstrated through structured, processes and systems designed to facilitate research [33]. - Advocate for continued organisational commitment to research [9]. - Increase awareness and understanding of the value of evidence-based practice [9]. - Commitment of senior clinical managers on research steering groups, which helps establish a research culture leading to organisational change) [41]. - Need high level support from professional and strategic managers in order to facilitate the greatest culture change in a sustainable way, e.g. through the legitimisation of research practices in the workplace, identification of mechanisms for supporting individuals and/or teams to undertake research [41]. - Develop a broad-based definition of research to legitimise a range of activities such as audits, journal clubs/groups, workplace publications and formal research activities within usual practice [41]. - High level support from management to legitimise research practices in the workplace [41].   Research as core business   - Recognise that building research capacity is useful because the skills of critical thinking can also be applied to practice decision making, which supports quality improvement approaches [29]. - Prioritise research projects which will generate knowledge which is relevant to service user and practice concerns, and able to inform clinical decision-making in practice [29]. - Help plan, design and implement projects which use patient-centred outcome measures in projects and realistic methodologies that are feasible in practice [29]. - Research needs to be considered as core business, built into the expectation of what everyone does [33]. - Ensure that research is built into the organisation’s missions and values [33]. - Team leaders and managers value and prioritise research as being part of core practice, built into the expectation of what everyone does [33]. - Ensure that research is built into the organisation’s missions and values [33]. - Explicitly label research activities as enhancing research consciousness among staff [41]. - Team-based research strategy/planning meetings (to contribute to individuals’ awareness of their position within a research culture) [41]. - Drive growth in the research agenda [9]. - Team leaders encourage discussions about research and evaluation during staff forums and team discussions [40]. - Research is added as an item for discussion in regular meeting agendas [40]. - Keep research issues a factor in daily practice and program planning [40]. - Research written into Role Descriptions (e.g. for certain positions) – to reinforce research as a core skill and activity, and to allow it to be reviewed as part of annual performance appraisals [29]. - Research as part of staff performance appraisals / professional development plans – reviewed in annual performance appraisals [29]. - Research written into job description of all, or at least some, staff (especially senior staff) [33]. - Expect and encourage research participation among staff [40]. - Include research in staff position descriptions [40]. - Include research in the orientation of new staff members [40].   Prioritisation of research that is ‘close to’/relevant to practice   - The underlying philosophy for developing research capacity in health is that it should generate research that is useful for practice, to improve the health of patients [29]. - Prioritise and support research projects which will generate knowledge which is relevant to service user and practice concerns, hence more likely to be taken up in practice to inform clinical decision-making [29]. - Research questions should be generated by, or in consultation with, practitioners and service users [29]. - Identify research priorities based in gaps in knowledge at a team/organisational level [29]. - Develop research questions in line with practice needs and priorities [29]. - Support and lead the design of research projects that use patient-centred outcome measures [29]. - Use action-oriented research methodologies [29]. - Identify strategic drivers for research [41]. - Support small scale projects that strategically address local clinical and service needs [41]. - Specify measurable outcomes and impacts of research projects for practice at the outset, and link these to the strategic aims of the team and organisation [41]. - Support research projects which are strategically important to the team/service (in line with the team’s strengths and priority areas) – i.e. local clinical and service needs [41]. - Systematically solicit and develop research questions and ideas that arise directly from practice [41]. - Focus on increasing capacity for research that is relevant to clinical practice in the public health sector [9].   Integration of local research findings back into practice   - Create opportunities to disseminate research findings locally and widely [29]. - Support and encourage dissemination of research findings using a range of methods including journal articles, conferences, local reporting seminars, lay publications, fact sheets, media, to ensure that the research has an impact on practice and local strategy/policy [29]. - Apply locally developed research knowledge to inform strategy policy [29]. - Encourage action research and participatory inquiry – e.g. cycles of action, reflection and dissemination, whereby use of research findings is integral to the process [29]. - Management co-presenting research findings to staff forums, the Board of Management, CEOs of other services etc [40]. - Showcase examples of quality research [9]. |
